# Complete mitogenomes of the chlorophycean green algae *Bulbochaete rectangularis* var. *hiloensis* (Oedogoniales) and *Stigeoclonium helveticum* (Chaetophorales) provide insight into the sequence of events that led to the acquisition of a reduced-derived pattern of evolution in the Chlamydomonadales and Sphaeropleales

**DOI:** 10.1080/23802359.2019.1710607

**Published:** 2020-01-14

**Authors:** Monique Turmel, Anne-Sophie Bélanger, Christian Otis, Claude Lemieux

**Affiliations:** Département de biochimie, Institut de Biologie Intégrative et des Systèmes, Université Laval, Québec, Canada

**Keywords:** Chlorophyceae, cox2a, fragmented rRNA genes, reduced gene content, genetic code

## Abstract

Mitogenome evolution in the Chlorophyceae is characterized by the acquisition of a reduced-derived pattern by the Chlamydomonadales + Sphaeropleales clade. Because no mitogenomes are available for the sister clade Oedogoniales + Chaetophorales + Chaetopeltidales, it remains unclear whether the common ancestor of chlorophycean green algae harbored a reduced-derived or ancestral-type mitogenome. The 70,191 and 46,765-bp mitogenomes reported here for *Bulbochaete rectangularis* var. *hiloensis* (Oedogoniales) and *Stigeoclonium helveticum* (Chaetophorales), respectively, shed light on this question. Both contain the same set of 41 conserved genes, a repertoire lacking numerous protein-coding genes but featuring all 27 tRNA genes typically found in ancestral-type mitogenomes.

The Chlorophyceae is a late-diverging group of chlorophyte green algae that comprises the Chlamydomonadales (Volvocales) + Sphaeropleales (CS) clade and the Oedogoniales + Chaetophorales + Chaetopeltidales (OCC) clade (Turmel et al. 2008). Chlamydomonadalean mitogenomes are characterized by derived traits, including a highly reduced gene content (12 genes), discontinuous rRNA genes (*rns* and *rnl*) occurring as multiple and dispersed fragments throughout the genome, a nonstandard genetic code, and high sequence divergence (Smith et al. 2013). Although their gene repertoire (37–42 genes) underwent less erosion (Nedelcu et al. 2000; Fucikova et al. 2014), sphaeroplealean mitogenomes also show multiple sites of rRNA gene fragmentation and nonstandard genetic codes (Noutahi et al. 2019; Zihala and Elias 2019) and in addition encode derived tRNA genes. To gain better insight into how the mitogenome was transformed during evolution of the Chlorophyceae, we sequenced the mitogenomes of *Bulbochaete rectangularis* var. *hiloensis* (Oedogoniales) and *Stigeoclonium helveticum* (Chaetophorales).

*Bulbochaete rectangularis* (UTEX LB 954) and *S. helveticum* (UTEX 441) were obtained from the Culture Collection of Algae at the University of Texas at Austin. An organelle DNA fraction of the former alga was submitted to 454 pyrosequencing and the resulting reads were assembled using Newbler v2.5 (Margulies et al. 2005). Sanger sequencing of the *S*. *helveticum* mitogenome was performed using plasmid clones derived from an organelle DNA fraction as detailed in (Belanger et al. 2006). Sequence data were generated by the Genomic Analysis Platform of Laval University and analyzed as described in (Turmel et al. 2010).

The 70,191-bp mitogenome of *B*. *rectangularis* (GenBank MN810331) and the 46,765-bp mitogenome of *S. helveticum* (GenBank MN810332) contain the same set of 41 conserved genes, which includes *cox2a* (a partial version lacking about 100 codons) and 11 other respiratory protein-coding genes that are all shared with Sphaeropleales (only *atp6* is missing). In contrast, there exist notable differences in tRNA gene composition between the OCC clade and Sphaeropleales. The newly reported algal mitogenomes use a standard genetic code and their 27 tRNA genes all have counterparts in ancestral-type mitogenomes of chlorophytes (Turmel et al. 1999; Turmel, Otis, de Cambiaire, et al. 2020; Turmel, Otis, Lemieux 2020). While the *rns* genes of both algae and the *B*. *rectangularis rnl* are continuous, the *S. helveticum rnl* occurs as two separate pieces, with the breakpoint coinciding with a fragmentation site shared by Chlamydomonadales and Sphaeropleales (L2/L3 junction in Sphaeropleales). The *B*. *rectangularis* and *S. helveticum* mitogenomes exhibit 18 and 13 introns, respectively.

Phylogenetic analysis of concatenated proteins using RAxML v.8.2.3 (Stamatakis 2014) recovered the two newly sampled chlorophyceans as a strongly supported clade with short branches at a position sister to the Sphaeropleales (Figure 1).

**Figure 1.**
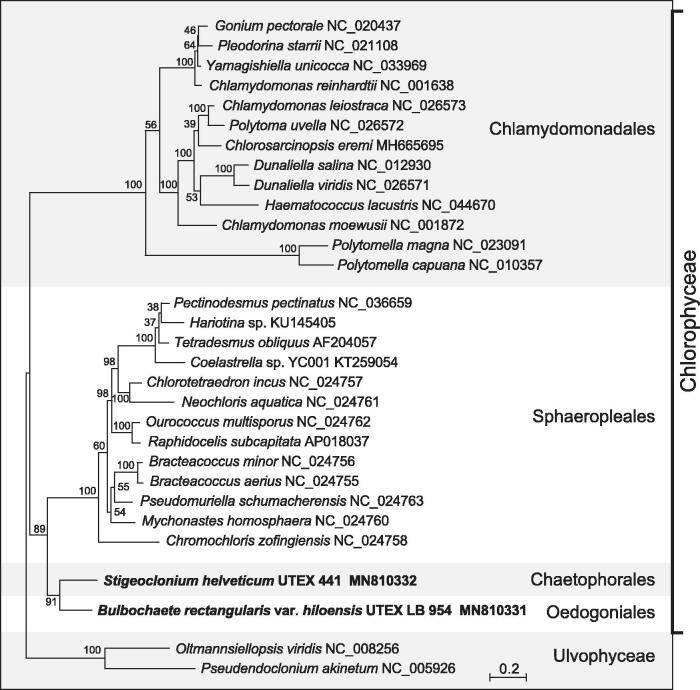
RAxML analysis of 13 concatenated mitogenome-encoded proteins from 30 chlorophytes. The figure shows the best-scoring tree, with bootstrap support values (100 replicates) reported on the nodes. GenBank accession numbers are provided for the mitogenomes of all taxa. The scale bar denotes the estimated number of amino acid substitutions per site. The data set was generated using the predicted protein sequences derived from *atp6*, *atp9*, *cob*, *cox1*, *cox2*, *cox3*, *nad1*, *nad2*, *nad3*, *nad4*, *nad4L*, *nad5*, *nad6*. Following alignment of the sequences of individual proteins with Muscle v3.7 (Edgar 2004), ambiguously aligned regions were removed using TrimAL v1.4 (Capella-Gutierrez et al. 2009) with the options block = 6, gt = 0.7, st = 0.005 and sw = 3, and the protein alignments were concatenated using Phyutility v2.2.6 (Smith and Dunn 2008). The phylogenetic analysis was carried out under the GTR + Γ4 model.

In conclusion, the mitogenome from the common ancestor of chlorophyceans possessed a reduced repertoire of protein-coding genes but its set of tRNA genes was complete, resembling those found in ancestral-type mitogenomes. Although its *rnl* gene was likely continuous, it might have encoded two distinct RNA species resulting from posttranscriptional cleavage at the sequence corresponding to the fragmentation site in *S*. *helveticum rnl*.
